# Exposure to lipopolysaccharide and calcium silicate-based materials affects the behavior of dental pulp cells

**DOI:** 10.1590/0103-6440202204990

**Published:** 2022-10-21

**Authors:** Marlus da Silva Pedrosa, Handially dos Santos Vilela, Juliana Garuba Rahhal, Natália Pieretti Bueno, Fabianne Soares Lima, Fernando Neves Nogueira, Carla Renata Sipert

**Affiliations:** 1 University of São Paulo- USP, School of Dentistry, Department of Biomaterials and Oral Biology, São Paulo, SP, Brazil.; 2 University of São Paulo- USP, School of Dentistry, Department of Restorative Dentistry, São Paulo, SP, Brazil.; 3 University of São Paulo- USP, School of Dentistry, Department of Oral and Maxillofacial Surgery, Prosthesis and Traumatology, São Paulo, SP, Brazil.

**Keywords:** Endodontics, dental cements, dental pulp, cytotoxicity tests, cytokines

## Abstract

This study assessed the cell viability, cytokine production, and mineralization potential of human dental pulp cells (hDPCs) after exposure to lipopolysaccharide (LPS) and application of calcium silicate-based materials (CSBM). Characterization of the CSBM was performed by infrared spectroscopy (n = 3). Extracts of Bio-C Repair, Biodentine, Cimmo HD, and MTA Repair HP were prepared and diluted (1:1, 1:4, and 1:16). Culture of hDPCs was established and treated or not with 1 µg/mL of LPS from *Escherichia coli* for 7 days. MTT assay was used to assess cell viability at 24, 48, and 72 h (n = 6). Alkaline phosphatase (ALP) activity was assayed on day 7 (n = 4). *Il-10* and *TNF-α* were quantified by ELISA at 24 h (n = 6). Data were analyzed by ANOVA and Tukey’s test (α = 0.05). Cell viability of LPS-activated hPDCs was higher than untreated control in 48 and 72 h (p < 0.05). Differences between non-treated and LPS-activated hPDCs were observed for Biodentine and Cimmo HP (p < 0.05). The CSBM influenced the cell viability (p < 0.05). ALP activity was higher in LPS-activated hDPCs (p < 0.05). No changes in the concentration of *TNF-α* were observed between groups (p > 0.05). The CSBM increased the *Il-10* production (p < 0.05). LPS-activated hDPCs presented increased cell viability and ALP activity. The CSBM showed mild toxicity and was able to enhance the cell viability and mineralization potential of untreated and LPS-activated hDPCs. The CSBM also induced anti-inflammatory mechanisms without compromising pro-inflammatory ones.

## Resumo

Este estudo avaliou a viabilidade celular, produção de citocinas e potencial de mineralização de células da polpa dentária humana (hDPCs) após exposição a lipopolissacarídeo (LPS) e aplicação de materiais à base de silicato de cálcio (CSBM). A caracterização do CSBM foi realizada por espectroscopia (n = 3). Extratos de Bio-C Repair, Biodentine, Cimmo HD e MTA Repair HP foram preparados e diluídos (1: 1, 1: 4 e 1:16). A cultura de hDPCs foi estabelecida e tratada ou não com 1 µg / mL de LPS de *Escherichia coli* por 7 dias. O ensaio de MTT foi usado para avaliar a viabilidade celular em 24, 48 e 72 h (n = 6). A atividade da fosfatase alcalina (ALP) foi avaliada no dia 7 (n = 4). Il-10 e TNF-α foram quantificados por ELISA em 24 h (n = 6). Os dados foram analisados ​​por ANOVA e teste de Tukey (α = 0,05). A viabilidade celular das hPDCs ativados por LPS foi maior do que o controle não tratado em 48 e 72 h (p <0,05). Diferenças entre hPDCs não tratados e ativados por LPS foram observados para Biodentine e Cimmo HP (p < 0,05). Os CSBM influenciaram na viabilidade celular (p <0,05). A atividade de ALP foi maior em hDPCs ativadas por LPS (p <0,05). Não foram observadas alterações na concentração de TNF-α entre os grupos (p> 0,05). Os CSBM aumentaram a produção de Il-10 (p < 0,05). Os hDPCs ativados por LPS apresentaram um aumento na viabilidade celular e atividade ALP. Os CSBM apresentaram toxicidade moderada e foram capazes de aumentar a viabilidade celular e o potencial de mineralização de hDPCs não tratados e ativados por LPS. Os CSBM também induziram mecanismos anti-inflamatórios sem comprometer os pró-inflamatórios.

## Introduction

The endodontic treatment aims to eliminate inflammatory and infectious processes and promote an adequate environment for tissue repair. Calcium silicate-based materials (CSBM) are commonly used in endodontics due to their ability to stimulate the healing of dental tissues by inducing cell proliferation, expression of osteogenic markers, and increasing mineralization processes. In addition, they orchestrate the crosstalk between immune response and tissue regeneration ^1^.

Human dental pulp cells (hDPCs) usually remain quiescent when they are within the dental pulps, but respond quickly after injury ^2^. These cells present a high capacity for proliferation, differentiation, and regeneration of dentin/pulp-like complex. Therefore, considering the interaction between bioactive materials and stem cells, dental pulp regeneration seems to be a promising strategy ^2^.

In pulp therapy, capping materials are more likely to be placed in contact with inﬂamed pulp tissues ^3^. Although there is a vast literature on the eﬀect of CSBM on dental pulp tissue or dental pulp cells, the majority of these studies were carried out without considering the inflammatory process caused by traumatic or bacterial injury ^3^.

This study aimed to investigate the cell viability, cytokine production, and mineralization potential of hDPCs after exposure to lipopolysaccharide (LPS) and application of calcium silicate-based materials. The results were used to test the null hypotheses: 1. the stimulation of the inflammatory process by LPS would not influence cell viability, cytokine production, and osteogenic potential of the hDPCs; 2. the application of the CSBM would not influence the cell viability, cytokine production and osteogenic potential of untreated and LPS-activated hDPCs.

## Material and Methods

This study was approved by the Ethics Committee of the School of Dentistry of the University of São Paulo (CAEE: 49499521.9.0000.0075, Protocol #4.881.363) and was conducted according to the Helsinki declaration.

### Attenuated total reflection in Fourier transform infrared spectroscopy (ATR-FTIR)

The materials were manipulated according to the manufacturer's instructions and inserted into a matrix designed for the production of specimens (7 mm x 1 mm, n = 3) and stored dry for 24 h (37°C). After this period, the specimens had their surface evaluated by mid-infrared spectroscopy (Vertex 70, Bruker Optics GmbH, Germany) using an attenuated full reflectance accessory (ATR, MIRacle, Pike Technologies, USA) with the diamond crystal. The spectra of the specimen were collected at three different points in the range between 400 cm^-1^ to 4,000 cm^-1^ at a resolution of 4 cm^-1^, using 64 scans per spectrum.

### Culture of hDPCs

The hDPCs were obtained from the biobank cell of the School of Dentistry of the University of São Paulo and cultured at standard conditions (37°C, 100% humidity, 5% CO2, and 95% air) in proliferation medium (PM): α-MEM (Invitrogen - Thermo Fisher Scientific, Waltham, MA, USA) with 10% fetal bovine serum (FBS) (Gibco - Thermo Fisher Scientific, Waltham, MA, USA) and antibiotics (100 µg/mL penicillin, 100 µg/mL streptomycin, 0.5 mg/mL amphotericin B - Invitrogen). hDPCs cells from passages four to eight were used for the assays ^4^.

### Extract preparation

All materials (Box 1) were manipulated and inserted into a round metal appliance designed for the production of specimens measuring 5 mm wide and 3 mm high. Specimens were allowed to be set for 24 h in a humid atmosphere and under aseptic conditions. After setting, each specimen was immersed into 1 mL of PM or osteogenic medium (OM) and incubated for 72 h ^4^. The OM was prepared by adding 100 nM dexamethasone, 10 mM β-glycerol-phosphate, and 0.05 mM 2-phosphate-ascorbic acid into the PM. The specimens were then discarded and a 0.22-µm pore size membranes (Millipore; Billerica, MA, USA) filtered the extracts. The extracts were diluted (1:1, 1:4, 1:16) in PM for MTT and ELISA assays and OM for alkaline phosphatase (ALP) activity, and stored at - 80 ºC until use ^4^.

**Box 1:**
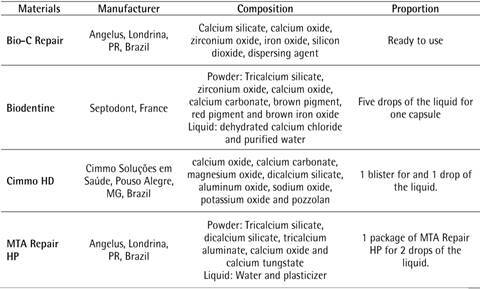
Tested materials

### LPS treatment

The hDPCs were primed or not with 1 µg/mL Escherichia coli LPS (L4391; Sigma-Aldrich, St Louis, MO, USA) for 7 days with medium change every other day. Next, cells were detached, counted, and seeded.

### Cell stimulation with materials extracts

The pure extract (1:1) was diluted (1:4 and 1:16) in α-MEM with 10% FBS. Untreated and LPS-activated hDPCs were counted and seeded at 2 ×10^4^ cells/well in 96-well plates in α-MEM with 10% FBS (n=9). After 24 h, the cells were incubated with 100 µL of the extracts or medium only (negative control group) ^4^.

### MTT assay

The untreated and LPS-activated hDPCs were counted and seeded at 2 ×10^4^ cells/well in 96-well plates in PM (n = 6). After 24 h, the cells were stimulated with the extracts for 24, 48, and 72 h. In the negative control group (NC), only PM was applied to the cells ^4^. The cell supernatant was replaced by 20 μL of a solution of 5 mg/mL of MTT [3-(4,5-Dimethylthiazol-2-yl)-2,5-diphenyltetrazolium bromide] (Sigma-Aldrich, St. Louis, MO, USA) in phosphate-buffered saline, followed by 180 μL of α-MEM with 10% FBS. Cells were incubated for 4 h and the MTT solution was replaced by 100 µL of dimethyl sulfoxide (Synth, Diadema, SP, Brazil). Optical density was determined at 570 nm.

### Alkaline phosphatase activity assay

The untreated and LPS-activated hDPCs were seeded in the 48-wells plate (2 × 10^4^ cells/well) and stimulated for 7 days with the extracts of the CSBM (n = 4) made in OM. Only OM was applied to the cells in the negative control group (NC). The medium was changed every two days. The ALP activity was measured by the colorimetric method of p-nitrophenyl phosphate (pNPP) using a kit (Labtest, Brazil). Briefly, the media were removed and 1% of sodium lauryl sulfate was added to each well. Then, 50 μL of the cell lysate, 50 μL of thymolphthalein monophosphate substrate, and 500 μL of buffer were mixed and kept for 10 min at 37°C. The absorbance at 590 nm was measured (Synergy HT, Biotek, Instruments, Inc. Winooski, VT, USA). ALP activity was normalized by the total protein content and expressed as μmol of thymolphthalein/h/mg of protein ^4^.

### Quantification of cytokines

The untreated and LPS-activated hDPCs (2 x 10^4^) and stimulated for 24 h with the CSBM (1:4 dilution) prepared in PM (n = 6). Quantification of TNF-α and IL-10 concentrations were performed in the cell culture supernatants by commercially available Duo-Set Enzyme-linked immunosorbent assay (ELISA) kits from R & D Systems ^4^.

### Statistical analysis

Normal data distribution was verified through the Shapiro-Wilk normality test and data were analyzed by ANOVA and Tukey’s test (α = 0.05). Data are presented as mean ± standard deviation. All statistical analyses were performed using GraphPad Prism 7.00 (GraphPad Software, Inc., CA, US).

## Results

### ATR-FTIR spectroscopy

**Figure 1:**
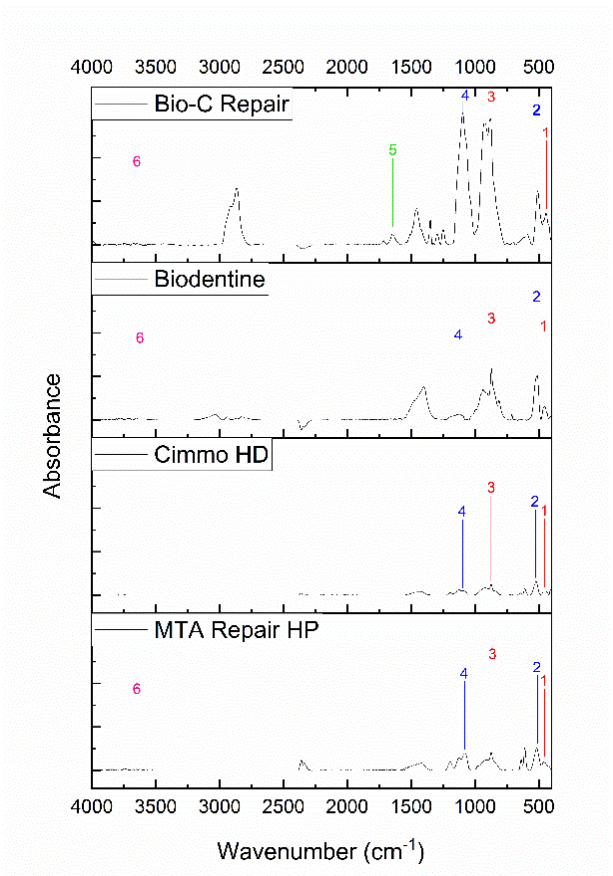
ATR-FTIR spectroscopy Bio-C Repair, Biodentine, Cimmo HD, and MTA Repair HP. The main peaks are highlighted.

Figure 1 shows the ATR-FTIR spectra of the CSBM. The intensity of the peaks in the FTIR-ATR analysis indicated the number of molecules present in the studied CSBM after setting ^5^ ^,^ ^6^. The more intense, the greater the presence of the vibrational mode. A correlation between the bands highlighted in Figure 1 and their reference peaks ^5^ ^,^ ^6^ is presented in Box 2.

**Box 2:**
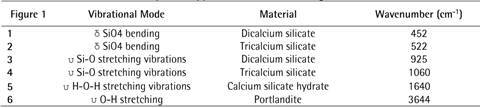
Fourier transform infrared spectroscopy information for the setting materials.

### MTT assay

**Figure 2:**
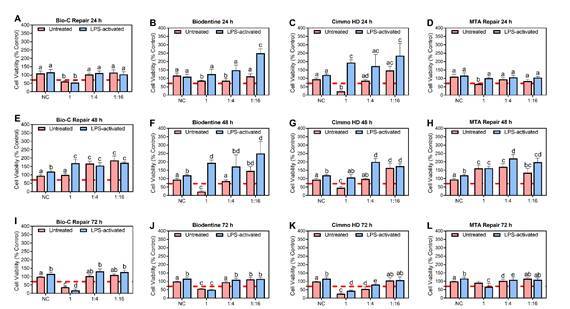
Heat map representing the cell viability rate (% of negative control group) according to MTT assay in hPDCs after 24 (A, B, C and D), 48 (E, F, G, and H ),and 72 (I, J, K and L), hours of exposure to different dilutions (1:1, 1:4 and 1:16) of the extracts of Bio-C Repair (A, E and I), Biodentine (B, F and J), Cimmo HD (C, G and K), and MTA Repair HP (D, H and L). hPDCs incubated in culture e medium alone served as the negative control group. hPDCs were seeded at a cell density of 2x10^4^ cells well in a 96-well culture plate. The results show the mean of the experiments (n = 9). Different letters represent signiﬁcant differences between groups. Two-Way ANOVA with Tukey test (p < 0.05). The horizontal dashed line indicates 70% cell viability. A decrease in cell viability below 70% of the control is considered cytotoxic according to ISO 10993-5.

A reduction in cell viability by more than 30% is considered cytotoxic according to ISO 10993-5:1999 ^7^. The cell viability of the LPS-activated hPDCs was higher than untreated control in 48 and 72 h (Figure 2). For Bio-C Repair, the pure extract at 24 (Figure 2A) and 72 h (Figure 2I) were found to be cytotoxic (p < 0.05). On the other hand, higher cell viability was observed for 1:4 and 1:16 dilution at 48 h (Figure 2E) (p < 0.05).

The pure extract of Biodentine at 48 h (Figure 2F) in untreated cells and 72 h (Figure 2J) in untreated and LPS-activated cells were cytotoxic (p < 0.05) while 1:16 dilution in 24 h and all dilutions in LPS-activated cells were higher than the negative control group (p < 0.05). Significant differences between non-treated and LPS-activated hPDCs were observed for all dilutions of Biodentine at 24 (Figure 2H) and 72 h (Figure 2J) (p < 0.05).

The pure extract of Cimmo HP was found to be mostly cytotoxic at 24 (Fig. 2C), 48 (Figure 2G) and 72 h (Figure 2K) (p < 0.05). 1:4 and 1:16 dilution, however, led to higher cell viability at 24 (Figure 2C) and 48 h (Figure 2G) (p < 0.05). LPS-activated hPDCs presented higher cell viability than untreated cells (p < 0.05).

No significant cytotoxic effect was noticed for MTA Repair HP (Figures 2D, 2H and 2L) (p > 0.05). All dilutions for this material at 48 h (Figure 2H) presented higher cell viability compared to the negative control group (p < 0.05).

### Alkaline phosphatase activity

**Figure 3:**
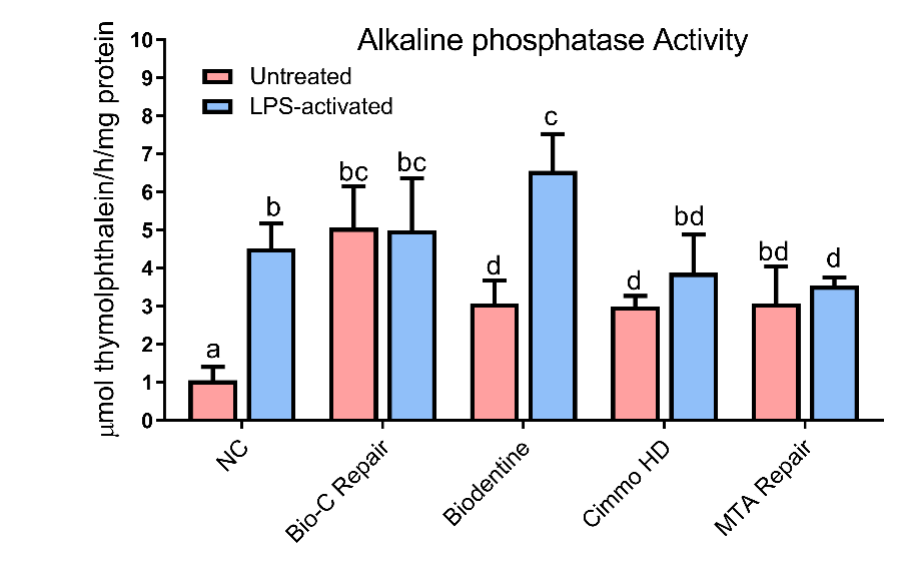
ALP activity after 7 days of exposure to 1:16 dilution of the extracts of Bio-C Repair, Biodentine, Cimmo HD, and MTA Repair HP in untreated and LPS-treated hPDCs. hPDCs incubated in a culture medium alone served as the negative control. The results show the mean and standard deviation of the experiments (n = 4). Different letters represent signiﬁcant differences between groups. Two-Way ANOVA with Tukey test (p < 0.05).

The activity of ALP (Figure 3) was higher in the LPS-activated hPDCs compared to the untreated control (p < 0.05). In untreated cells, the materials were able to induce a significant increase in ALP activity (p < 0.05). In the LPS-activated hPDCs, the application of MTA Repair HP caused a decrease in ALP activity (p < 0.05) which was still similar to the untreated cells for this same material (p < 0.05). Interestingly, a significant increase in ALP activity was noticed when this CSBM was applied in the LPS-activated hPDCs compared to untreated ones.

### Cytokine production

**Figure 4:**
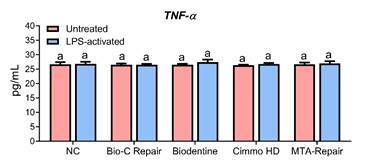
*TNF-α* concentration according to ELISA assay after 24 h of exposure to 1:4 dilution of the extracts of Bio-C Repair, Biodentine, Cimmo HD, and MTA Repair HP in untreated and LPS-treated hPDCs. hPDCs incubated in ca ulture medium alone served as the negative control. The results show the mean and standard deviation of the experiments (n = 6). Different letters represent signiﬁcant differences between groups. Two-Way ANOVA with Tukey test (p < 0.05).

**Figure 5:**
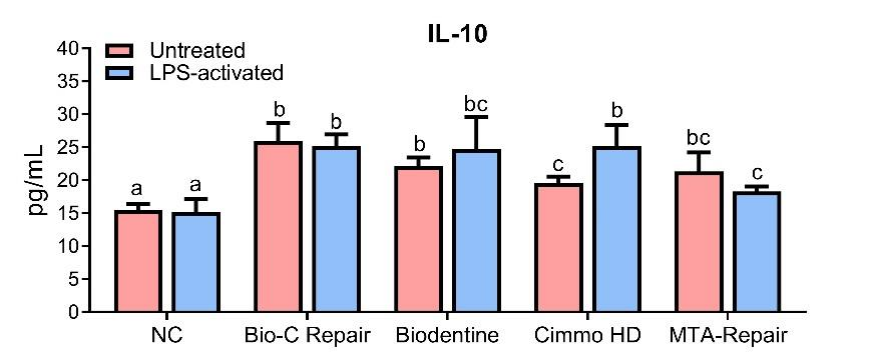
*Il-10* concentration according to ELISA assay after 24 h of exposure to 1:4 dilution of the extracts of Bio-C Repair, Biodentine, Cimmo HD, and MTA Repair HP in untreated and LPS-treated hPDCs. hPDCs incubated in a culture medium alone served as the negative control. The results show the mean and standard deviation of the experiments (n = 6). Different letters represent signiﬁcant differences between groups. Two-Way ANOVA with Tukey test (p < 0.05).

No changes in the concentration of *TNF-α* (Figure 4) were observed between groups (p > 0.05). Contrastingly, the application of CSBM significantly increased the *Il-10* production (Figure 5) by hDPCs (p < 0.05). Only for Cimmo HD, higher *Il-10* production was observed in the LPS-activated group compared to untreated ones (p < 0.05).

## Discussion

To promote the regeneration of mineralized tissues, an ideal biomaterial should lead to cell proliferation, differentiation and deposition, and mineralization of the extracellular matrix ^8^. These materials should also have a positive outcome in inflammation ^8^. In this study, the null hypotheses tested were rejected. The treatment with LPS showed significant effects on cell viability and ALP activity of hDPCs. The cell viability, *Il-10* production, and ALP activity were influenced by the application of CSBM.

Calcium silicate-based materials are di- and tricalcium silicates, which will react with water, and the final product will be a hydrated part (calcium silicate hydrate) and an anhydrate part (calcium hydroxide) ^5^ ^,^ ^6^. The intensity of the peaks in the FTIR-ATR analysis indicates the number of molecules present in the CSBM after the setting. The more intense, the greater the presence of the vibrational mode. Higher intensity peaks were observed for Bio-C Repair followed by Biodentine, MTA Repair HP, and Cimmo HD. The highest vibrational modes may indicate a higher amount of molecules available. Bio-C Repair was the only material that presented the peak characteristic of the hydrated phase of CSBM suggesting that the setting reaction was not fully completed for the other CSBM. This might lead to higher solubility and leaching of its components into the extract and consequently influence the outcomes investigated herein.

Lipopolysaccharide, the main component of the outer membrane of Gram-negative bacteria, is a potent stimulator of inflammation in hDPCs. LPS-induced inflammation causes changes in the adhesion, migration, proliferation, and differentiation of hDPCs ^9^ ^,^ ^10^. In this study, the hDPCs were treated with 1 μg/mL of LPS. This concentration simulates an inflamed microenvironment of dental pulp in a clinical scenario ^10^.

In this study, cell viability was assessed through an MTT assay. Even though this method measures metabolic activity and does not differentiate cell cycle, anti-proliferative effects, cell apoptosis, or necrosis, It is a reliable and accessible method to assess the cell viability of endodontic materials ^4^. We also worked with the CSBM extracts to infer a possible dose dilution that occurs in vivo ^4^ as well as the effects of these materials at a distance ^11^.

Calcium silicate-based materials enhance the proliferation of hDPCs probably due to the release of bioactive ions ^12^. In this study, the pure extract of all CSBM presented a cytotoxic effect at a certain point. Notably, the materials were also able to stimulate the viability of the hDPCs. Besides to the best of our knowledge, this is the first study evaluating the Cimmo HD, this increase in cell viability in untreated cells was already reported for the other tested materials in different cell lines ^13^ ^,^ ^14^ ^,^ ^15^ ^,^ ^16^ ^,^ ^17^ ^,^ ^18^. Interestingly, the increase in cell viability was even higher in the LPS-activated hDPCs. As also reported herein, Biodentine promotes proliferation in both normal and LPS-induced DPCs ^19^. This might suggest that the activation with LPS, which simulated an inflammatory environment (pulpitis), may have a positive effect on the proliferative capacity of hDPCs.

The alkaline phosphatase is highly expressed in cells of mineralized tissues playing a critical function in the formation of hard tissue. Thus, an increased level of ALP activity would favor tissue repair ^20^. Increased ALP activity, which was observed in hDPCs treated with LPS ^9^, was also correlated with inflammation and further healing ^21^. Literature shows that CSBM stimulates hDPCs differentiation and mineralization by activating the MAPK pathway ^22^. In this study, treatment with LPS and the application of the CSBM increased the ALP activity. This might suggest that the materials induce a mild inflammation and have a positive effect on mineralization potential. Furthermore, these data should be complemented by future *in vitro* and *in vivo* studies with a deeper evaluation of the osteogenic potential of these CSBM.

Cytokines are molecular messengers that promote inflammatory and wound healing events ^23^. Treatment with LPS is known to cause increased expression of several pro-inflammatory cytokines ^24^. The CSBM elute ions that stimulate the production of different pro-inflammatory and anti-inﬂammatory cytokines that contribute to the healing of the dental pulp ^3^.

Biodentine did not induce a significant inflammatory process in rat subcutaneous tissues ^25^. In this study, no changes in *TNF-α* quantification in LPS-activated hDPCs were observed, which is in agreement with the literature ^1^. In addition, as observed here, CSBM neither induced nor aggravated LPS-induced inflammation ^19^.

In this study, *Il-10*, an anti-inflammatory cytokine, was increased for all CSBM. Taken collectively, these results showed that the CSBM tested were able to induce anti-inflammatory mechanisms, which is of great interest for clinical practice. This was reported in mouse bone marrow mesenchymal stromal cells ^1^. To the best of our knowledge, literature still lacks studies evaluating the production of these cytokines considering the previous inducement of the inflammatory response of hDPCs by LPS.

Besides, there is strong evidence showing that CSBM increases the cell viability of hDPCs, it remains unclear how these materials induce healing and regeneration in inflamed human pulps ^19^. Thus, the results reported herein may shed a light on how these materials contribute to a better outcome in endodontic treatment.

## Conclusion

Human dental pulp cells treated with lipopolysaccharide presented increased cell viability and alkaline phosphatase activity.

The calcium silicate-based materials presented mild toxicity and were able to enhance the proliferation and mineralization potential of untreated and LPS-activated hDPCs.

The materials also induced anti-inflammatory mechanisms without compromising pro-inflammatory ones.
